# Genome-Wide Identification and Expression Profile of *Farnesyl Pyrophosphate Synthase* (*FPS*) Gene Family in *Euphorbia Hirta* L.

**DOI:** 10.3390/ijms26020798

**Published:** 2025-01-18

**Authors:** Xinyi Bian, Tingkai Wu, Runrun Qiang, Zhi Deng, Fazal Rehman, Qiyu Han, Dong Xu, Yuan Yuan, Xiaobo Wang, Zewei An, Wenguan Wu, Huasen Wang, Han Cheng

**Affiliations:** 1 College of Horticulture Science, Zhejiang Agriculture and Forestry University, Hangzhou 311300, China; bxyabcd2024@163.com (X.B.); whsych66@163.com (H.W.); 2 State Key Laboratory of Tropical Crop Breeding, Sanya Institute, Rubber Research Institute, Chinese Academy of Tropical Agricultural Sciences, Sanya 572025, China; qrrsicau@163.com (R.Q.); zizip@163.com (Z.D.); fazlpbg@gmail.com (F.R.); hanqiyu163@163.com (Q.H.); xudongzhuanyong@163.com (D.X.); yuanyuan_2023@126.com (Y.Y.); xiaobo_wang15@126.com (X.W.); anzewei@163.com (Z.A.); wenguanwu88@163.com (W.W.); 3 Rubber Research Institute, Chinese Academy of Tropical Agricultural Sciences, Haikou 571100, China

**Keywords:** Farnesyl diphosphate synthase, *Euphorbia hirta* L., bioinformatics, subcellular localization, expression analysis

## Abstract

The biosynthesis of isopentenyl diphosphate (IPP) and dimethylallyl diphosphate (DMAPP), which are essential for sesquiterpenes and triterpenes, respectively, is primarily governed by the mevalonate pathway, wherein *farnesyl pyrophosphate synthase* (*FPS*) plays a pivotal role. This study identified eight members of the FPS gene family in *Euphorbia hirta*, designated *EhFPS1*–*EhFPS8*, through bioinformatics analysis, revealing their distribution across several chromosomes and a notable tandem gene cluster. The genes exhibited strong hydrophilic properties and key functional motifs crucial for enzyme activity. An in-depth analysis of the *EhFPS* genes highlighted their significant involvement in isoprenoid metabolism and lipid biosynthesis, with expression patterns influenced by hormones such as jasmonic acid and salicylic acid. Tissue-specific analysis demonstrated that certain *FPS* genes, particularly *EhFPS1*, *EhFPS2*, and *EhFPS7*, showed elevated expression levels in latex, suggesting their critical roles in terpenoid biosynthesis. Furthermore, subcellular localization studies have indicated that these proteins are primarily found in the cytoplasm, reinforcing their function in metabolic processes. These findings provide a foundational understanding of the *FPS* genes in *E. hirta*, including their gene structures, conserved domains, and evolutionary relationships. This study elucidates the potential roles of these genes in response to environmental factors, hormone signaling, and stress adaptation, thereby paving the way for future functional analyses aimed at exploring the regulation of terpenoid biosynthesis and enhancing stress tolerance in this species.

## 1. Introduction

*Euphorbia hirta* L. is a common herbaceous plant, also known as a hairy spurge, asthma weed, snakeweed, or ara tanah, belonging to the genus *Euphorbia* in the family Euphorbiaceae. It is native to Central America, was later introduced to Southeast Asia, and is widely distributed in tropical and subtropical regions worldwide [1]. In China, it is mainly distributed in the south and southwest, including Hainan, Fujian, Guangdong, Guangxi, Guizhou, Yunnan, and Taiwan. It has a unique morphology, with an erect branched stem covered with brown or yellowish-brown coarse hairs; opposite leaves, oblong or lanceolate, with serrated lobes on the leaf margins; numerous inflorescences densely packed in the axils of the leaves; and triangular capsules covered with short, soft hairs [2]. *E. hirta* has a strong reproductive capacity and grows on grassy and shrubby hillsides, mostly in sandy soils [3].

As a traditional Chinese herbal medicine, the whole plant is rich in various active substances, including polyphenolic flavonoids such as luteolin, quercetin, 3′-methylmyricetin, quercetin, and gallic acid [4]; tannins such as protocatechuic acid, tannic acid, protochebulic acid, chlorogenic acid, and 3,4-di-o-galloylquinic acid; and terpenoids and triterpenoids such astaraxacin,23–25-methoxycyclophane-3-ol,tinyatoxin-12-deoxy-4β-Hydroxyphorbol-13-dedocanoate-20-aceate, and 12-deoxy-4β-hydroxyphorbol [5,6]. These compounds provide the herbs with unique medicinal properties. Modern pharmacological studies have shown that E. hirta has antibacterial, anticancer, anti-inflammatory, antiasthmatic, and diuretic effects [7]. It is mainly used to treat lung abscess, furuncle, dysentery, diarrhea, stranguria, hematuria, eczema, tinea pedis, skin itching, postpartum low milk production, and other diseases [8]. Terpenoids are compounds derived from mevalonate with isoprene units as the basic structural unit of the molecular skeleton and their extensions. This process involves multiple enzyme catalyses and complex regulatory networks.

Farnesyl pyrophosphate synthase (FPS) is a key enzyme that regulates the synthesis of farnesyl diphosphate (FPP) from isopentenyl pyrophosphate (IPP) and dimethylallyl diphosphate (DMAPP) via the mevalonate pathway (MVA). Its catalytic synthesis products are precursors of various terpenoid derivatives such as steroids, saponins, sesquiterpenes, and triterpenes in plants [9]. Therefore, because of their link to an important rate-limiting enzyme in the terpene synthesis pathway, cloning and functional analysis of *FPS* genes has always been a research hotspot. Researchers have cloned and identified farnesyl pyrophosphate synthase in many plants, including rubber trees, *Eucommia ulmoides*, *Panax notoginseng*, *Arabidopsis thaliana*, and tobacco [10,11]. The functional loss of *AtFPS1* and *AtFPS2* in *A. thaliana* can cause plant death [10].

It was also found that the content of carotenoids and terpene aroma substances in the leaves of transgenic FPS tobacco lines were significantly increased compared with the wild type [11]. Another study revealed heterologous expression of the *FPS* gene in tobacco, with significantly enhanced resistance to brown star and black neck diseases [12]. It was also identified that *EuWRKY30* can directly bind to the W-box element in the EuFPS1 promoter and activate its expression. In addition, overexpression of *EuWRKY30* significantly upregulates the expression of *EuFPS1*, further increasing the density of rubber particles and the content of Eucommia [13]. Using the yeast single hybrid screening library method, the upstream transcriptional regulatory factors GISNF2 and GIMHR of the *FPS* gene in the triterpene synthesis pathway of *Ganoderma lucidum* were identified, providing a basis for further investigation into the expression and regulation mechanism of the *FPS* gene [14]. However, to date, no genes in the *E. hirta FPS* gene family have been identified or systematically studied.

In this study, full-length sequences of three *FPS* genes from rubber trees were used as references. Eight EhFPS proteins were identified in the *E. hirta* genome by BLASTP. Bioinformatic and expression pattern analyses were performed to lay a theoretical foundation for further exploring the function of *EhFPS* and analyzing the synthesis pathways of hemiterpenes and triterpenes in *E. hirta*.

## 2. Results

### 2.1. Identification and Physicochemical Properties of EhFPS

The FPS protein domains in the rubber tree were identified using the conservative structure databases InterPro and CDD, and BLASTP comparison was performed in the constructed protein sequence library of *E. hirta*. Resultantly, eight *FPS* candidate genes, namely *EhFPS1, EhFPS2, EhFPS3, EhFPS4, EhFPS5, EhFPS6, EhFPS7*, and *EhFPS8*, were found as per position sequence in the *E. hirta* genome (Table 1). Analysis of the physicochemical properties of EhFPS proteins showed that the encoded amino acid lengths ranged from 323 to 389 aa, and the molecular weight of the protein was between 37.44 and 45.24 kDa. The smallest member was EhFPS5, consisting of 323 amino acids, with a molecular weight of 37.44 kDa, whereas the largest member was EhFPS3, consisting of 389 amino acids, with a molecular weight of 45.24 kDa. The isoelectric points were between 5.11 and 6.52, with mostly acidic proteins, and the average hydrophobicity index (AHI) was less than 0, ranging from −0.353 to −0.255, indicating that EhFPS proteins were all hydrophilic. In the secondary structure of the EhFPS protein, the highest proportion was the α-helix, and 75% of the proteins had an α-helix index greater than 60%; EhFPS5 had the highest index of 65.94%, and EhFPS4 had the lowest index of 56.72%. β-turn had the lowest percentage, with EhFPS8 accounting for the highest percentage (3.76%). Subcellular localization analysis showed that except for EhFPS3, EhFPS4, and EhFPS8, which were localized in chloroplasts, the others were localized in the cytoplasm.

### 2.2. Chromosomal Location and Gene Duplication Analysis of EhFPS

**Figure 1:**
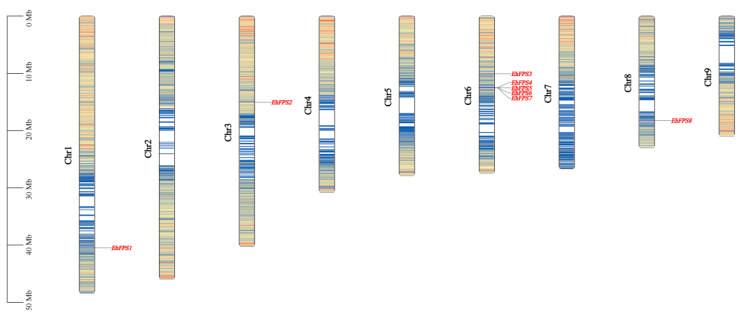
Genetic map of *E. hirta* and chromosome mapping of *FPS* genes on *E. hirta* genome. The scale to the left is in megabase units (Mb). The chromosome number is indicated to the left of each chromosome. Red-colored letters indicate gene positions on each chromosome. Blue lines on chromosomes indicate gene density. The greater the density of genes, the denser the blue line area.

**Figure 2:**
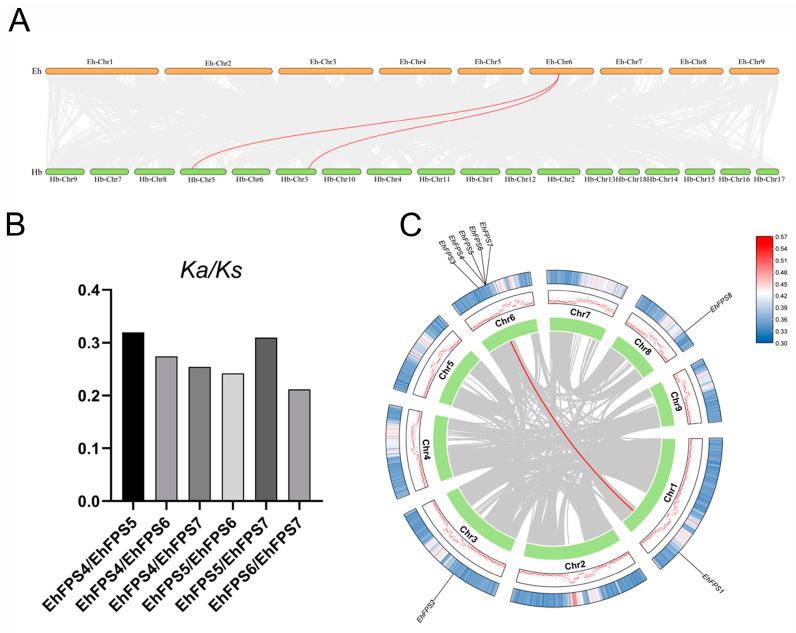
Series replication and selection pressure analysis of *FPS* genes. (**A**) Collinearity analysis of *FPS* genes between *E. hirta* and *H. brasiliensis*. Gray lines in the background highlight collinear blocks within the *E. hirta* and other plant genomes, while collinear *FPS* gene pairs are linked with red lines. (**B**) Selection pressure analysis of *FPS* gene cluster. (**C**) Analysis of tandem replication of *FPS* genes in the genome of *E. hirta*. Chromosomes 1~9 are represented by rectangles. Lines and histograms along the rectangle represent the gene density on the chromosome. The gray lines represent the isolinear blocks in the genome of *E. hirta*, while the red lines between chromosomes represent the gene pairs with fragment repeats.

To reveal the distribution of the *FPS* genes, their sequences were aligned to the genomic data of *E. hirta*, and chromosome mapping was performed using gene location information, as shown in Figure 1. These genes were unevenly distributed on the four chromosomes, with one member each on Ch1, Ch3, and Ch8. The number of *EhFPS* genes in Ch6 was the highest, with five members. A tandem gene cluster comprising *EhFPS4* and *EhFPS7* was identified on chromosome 6 within a 1 Mb region, along with fragment replication involving *EhFPS1* (Figure 2A). Evolutionary analysis of these tandem repeat genes revealed that their Ka/Ks ratio was significantly less than 1 (Figure 2B). A low Ka/Ks ratio suggests that nonsynonymous mutations are largely excluded, likely because of their detrimental effects on protein function. This phenomenon is typically observed in functionally important and conserved genes such as those encoding metabolically critical enzymes or structural proteins. Furthermore, analysis of gene duplication events indicated that the *FPS* genes of both *E. hirta* and rubber trees contained a pair of duplicate genes. Specifically, *EhFPS4* on Ch6 in *E. hirta*, *EhFPS1* on Ch3, and *EhFPS2* on Ch5 in *Hevea brasiliensis* were identified as fragment repeat genes (Figure 2C). These findings suggest that homologous gene pairs emerged through gene replication during evolution.

### 2.3. Conserved Domains and Gene Structural Analysis of EhFPS

**Figure 3:**
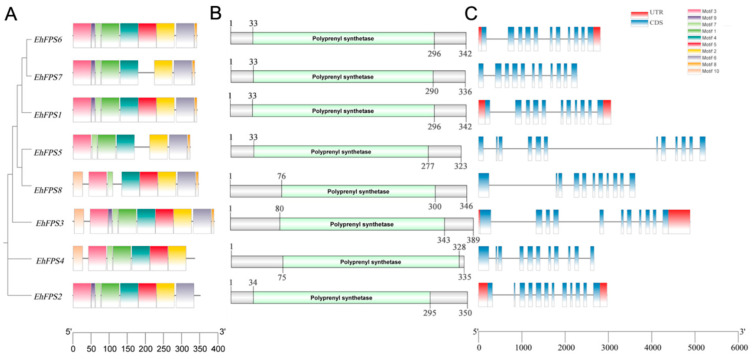
The sequence motif, conserved domains, and gene structural analysis of EhFPS. (**A**) The motif prediction of gene structures. In motif pattern, different colored boxes indicate different motifs; motif1–motif10 were represented by different numbers in the boxes (1–10). (**B**) Conserved domain prediction. The conserved structure of *FPS* genes with only one polyprene synthetase domain was predicted by NCBI-CDD, and the gray color represents the conserved structure of polyprene synthetase domain. (**C**) Gene structure map. The blue bar indicates the coding sequence (CDS), the black line indicates the intron, and the red bar indicates the untranslated region (UTR).

**Figure 4:**
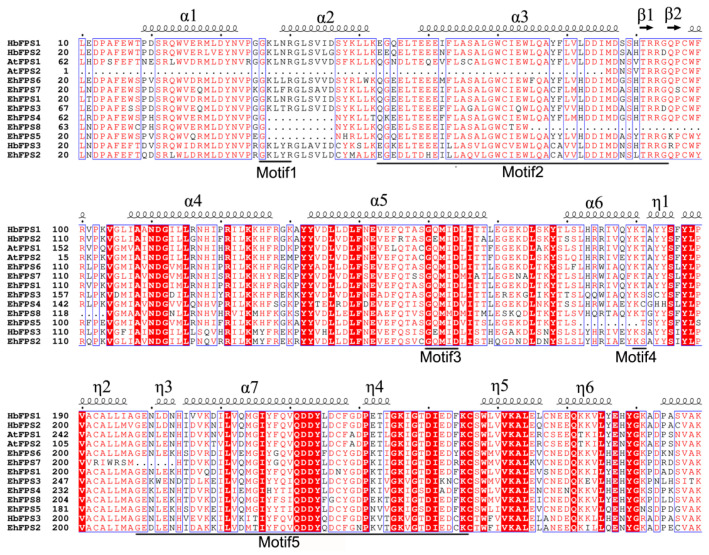
Multiple sequence alignment of a conserved region from *E. hirta*, *H. brasiliensis*, and *A. thaliana*. Motif1 (GKXXR), motif2 (EXXXXXXLXXDDXXDXXXXRRG), motif3 (GQXXD), motif4 (KT), and motif5 (GXXFQXXDDXXDXXXXXXXXGKXXXDXXXXK). Among them, the lysine residue of motif1 can promote the binding of FPPS with isopentenyl pyrophosphate (IPP). Motif2 contains the first aspartic-acid-rich region, DDXXD, which determines the length of the product chain. The amino acid residues in this region participate in the formation of the C-C bond between IPP and the allylic substrate and determine the length of the product chain. In motif3, among the three fixed conserved amino acids, G (1st position), Q (2nd position), and D (5th position) are all conserved in all plant FPPS sequences. Motif4 is a conserved amino acid in all plant FPPS sequences. Motif5 contains the second aspartic-acid-rich region, DDXXD. The marked helices represent the α-helices in the FPS protein, which play a role in forming the catalytic pocket or stabilizing the protein structure.

**Figure 5:**
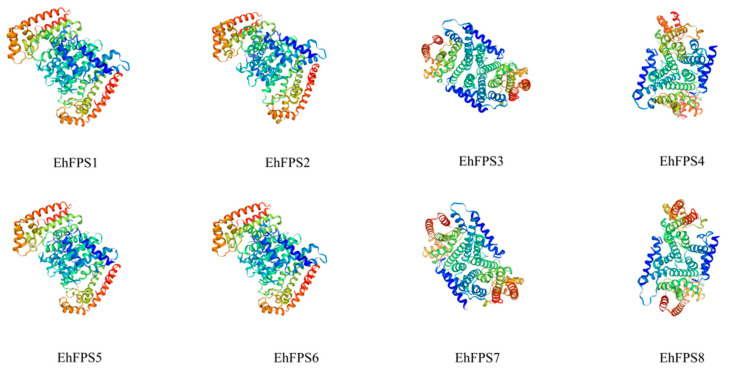
Modeling of eight EhFPS proteins. The structure of eight EhFPS proteins with >90% confidence level are shown. Using the SWISS-MODEL, the EhFPS protein sequence was used for modeling. The structures of eight EhFPS proteins with >90% confidence level are shown. The finest place in the figure is the loop region, which is divided into three channels: red, green, and blue. The range of each color channel is 0–255; 0 indicates that there is no color channel, and 255 indicates the maximum intensity of the color channel. The higher the channel value, the stronger the three colors.

Cluster analysis showed that *EhFPS* genes could be divided into three categories, of which *EhFPS2* and *EhFPS4* were clustered into one category, *EhFPS3* alone was in one category, and the remaining five members were in one category (Figure 3A). The conserved domains of each *EhFPS* gene were predicted using NCBI-CDD, and the results showed that each EhFPS protein contained a single polyprenyl synthase domain of a similar length (Figure 3B). The structures of *EhFPS* genes were similar and contained more exons; among them, *EhFPS2* and *EhFPS5* had 12 exons, and the remaining members had 11 exons. However, only *EhFPS6*, *EhFPS1*, *EhFPS3*, and *EhFPS2* had UTR ends, whereas the other four genes did not have UTR ends (Figure 3C). Comparing the conserved domains and motifs of all genes, except EhFPS8, the other seven members all contained the important functional domain motif II DDXXD, which binds Mg2+ to regulate enzyme activity, whereas Motif2 (DDYXD) was shared by all members (Figure 4). In addition, it contained GKLNR (Motif1), which binds to the substrate, and KT (Motif4), which is the active site of the enzyme. Through protein tertiary structure modelling, the sequence identity of the EhFPS protein and Eucommia farnesyl diphosphate synthase 1 chimera (template number: 7bux.1.A) from the PDB database (https://www.rcsb.org/, accessed on 7 May 2024) was 78.36%, the sequence similarity was 0.81, and the coverage was 0.86. Modelling based on this template showed that EhFPS is composed of homodimeric oligonucleotides formed by α-helices and that the spatial structure contains a “pocket” for binding to the substrate. At the same time, comparisons revealed that *EhFPS1, EhFPS2, EhFPS5*, and *EhFPS6* had seven α-helical chains with similar overall structures, *EhFPS3* and *EhFPS7* had similar side chain structures, and *EhFPS4* and *EhFPS8* had similar tertiary structures (Figure 5), indicating that changes in the protein structure of members may lead to protein functional diversity.

### 2.4. Sequence and Phylogenetic Analysis of Proteins Encoded by the EhFPS

**Figure 6:**
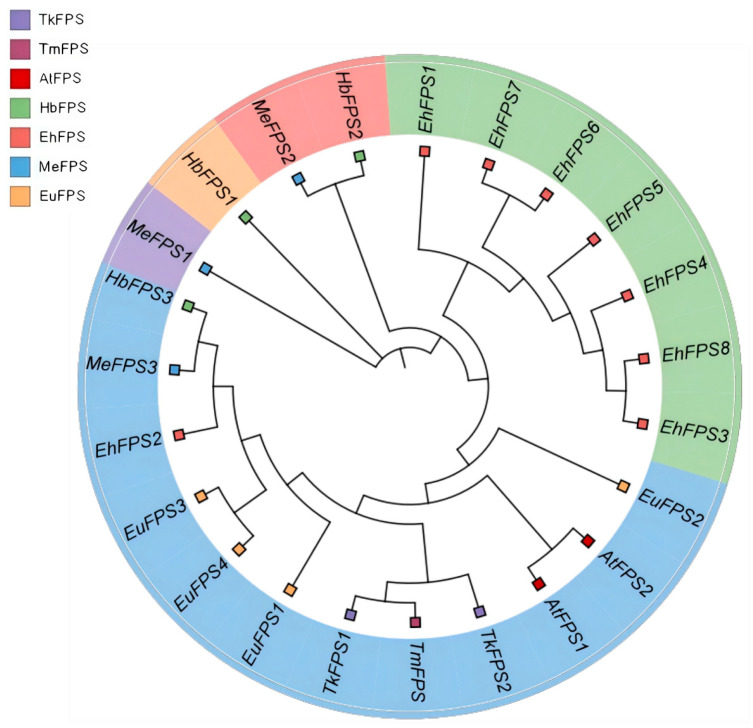
Phylogenetic tree of gene members of the FPS family in seven species. Squares of different colors represent *FPS* genes of different species. The five different subfamilies were distinguished by yellow and green bands.

To clarify the phylogenetic relationship between the *EhFPS* genes, the amino acid sequences of the *FPS* genes of *E. hirta*, rubber tree, cassava, *Eucommia ulmoides*, *Taraxacum koksaghyz* Rodin, *Taraxacum mongolicum*, and *A. thaliana* were used to construct a phylogenetic tree using the neighbor-joining method (Figure 6). *EhFPS* genes were divided into two subtribes, of which the branch of subtribe I contained all EhFPS proteins other than *EhFPS2*; *EhFPS1* was independently separated in this branch. The other subtribe in which *EhFPS2* was located also contained multiple proteins, such as *Taraxacum koksaghyz* Rodin, *Taraxacum mongolicum*, and *Eucommia ulmoides*, among which *EhFPS2* was closely related to *HbFPS3* in the rubber tree and *MeFPS3* in cassava. *MeFPS1* and *HbFPS1*, as well as *MeFPS2* and *HbFPS2*, were independently evolved subclades. In summary, there were homologous proteins in *Rhizoma Dioscoreae* that were closely related to the rubber tree, *Eucommia ulmoides*, and cassava, but most differentiated into independent branches. Therefore, it is speculated that it exercises a different functional mechanism from that of other plants such as rubber and cassava.

### 2.5. Gene Ontology and Interaction Network Analysis of EhFPS

**Figure 7:**
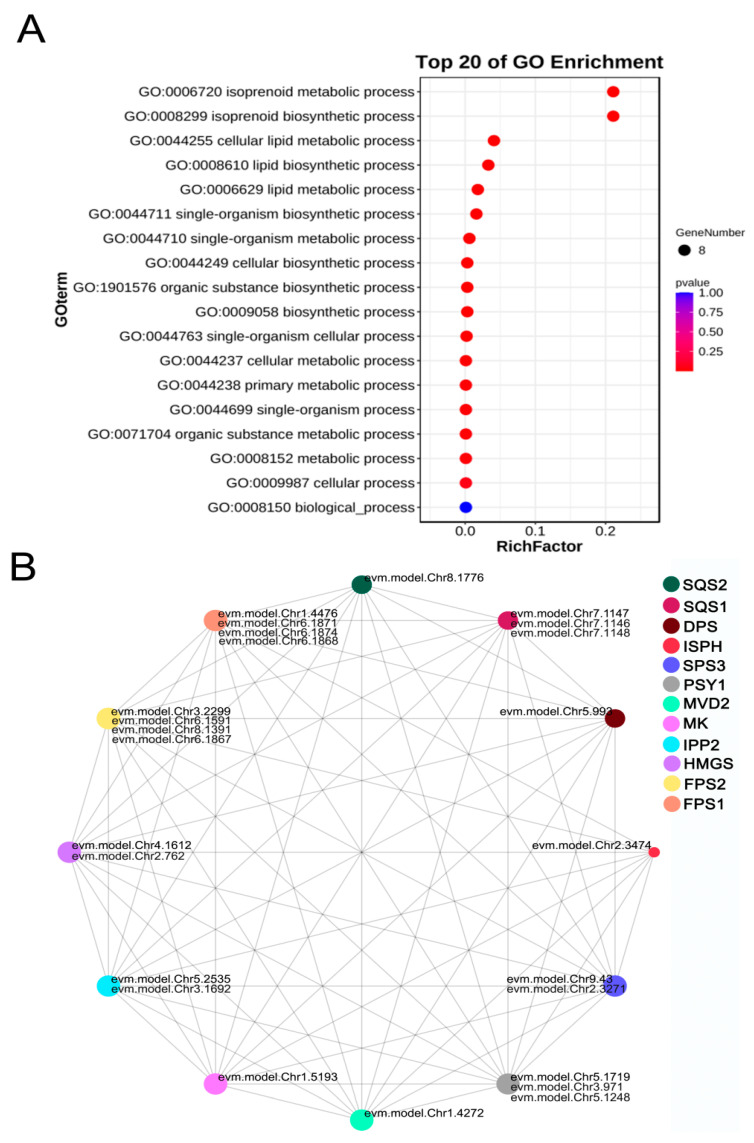
The gene ontology enrichment (GO) analysis and protein–protein interactions (PPI) of *EhFPS* genes. (**A**) GO analysis. The 20 enrichments with the highest scores were selected for display. The horizontal coordinate represents the gene ratio, and the circle represents the number of genes. (**B**) Protein interaction network of *E. hirta* genes mapped to *Arabidopsis* genes. The circle size indicates the number of interaction partners, with larger circles representing more extensive interaction networks.

Studies of *FPS* genes in various latex-producing plants have hypothesized that these genes are involved in a wide range of biological processes. GO enrichment analysis was conducted to investigate the functional roles of *FPS* genes in *E. hirta*; a gene ontology (GO) enrichment analysis was conducted (Figure 7A). The results revealed that eight genes were enriched in 18 GO terms (Table S1), all of which were categorized under biological processes. The top five significantly enriched terms were isoprenoid metabolic process (GO:0006720), isoprenoid biosynthetic process (GO:0008299), cellular lipid metabolic process (GO:0044255), lipid biosynthetic process (GO:0008610), and lipid metabolic process (GO:0006629). These findings suggest that *EhFPS* genes are likely to be involved in the synthesis and metabolism of isoprenoid compounds, as well as in lipid biosynthesis and metabolism. Overall, these results highlight the critical roles of *FPS* genes in the key physiological functions of *E. hirta*, including growth, development, stress adaptation, and maintenance of the cell membrane structure.

The protein-regulatory network of *A. thaliana* has been systematically studied as a model plant. FPS proteins were located in homologues of *A. thaliana*, and the interaction networks and their interaction networks with other proteins were deduced (Table S2). Farnesyl pyrophosphate synthases (FPS1 and FPS2) are key enzymes in the isoprene biosynthesis pathway in *A. thaliana*. They catalyze the condensation of isoprene pyrophosphate (FPP) from isoprene IPP and dimethylallyl pyrophosphate (DMAPP), which are direct precursors of isoprene compounds, such as carotenoids, sterols, and sesquiterpenes. In the protein–protein interaction (PPI) network maps constructed with FPS1 and FPS2, the protein showed extensive connections with multiple proteins, including upstream and downstream key enzymes, indicating that the FPS protein is not only the main producer of FPP, an intermediate product of the isoprene pathway, but the regulatory hub of the metabolic network. Among the proteins involved, HMGS, IPP2, and ISPH serve as upstream regulators of FPS, which utilizes IPP and DAMPP as substrates to directly influence metabolic flow in a manner dependent on HMGS and IPP2. Conversely, SQS1, SQS2, DPS, and PSY1 were identified as direct downstream proteins of FPS. The close association between FPS, SQS1, and SQS2 highlights their collaborative role in the sterol biosynthesis pathway. Specifically, SQS1 catalyzes the conversion of farnesyl pyrophosphate (FPP) to squalene, an intermediate product in the sterol biosynthesis pathway, and a precursor for the synthesis of gas triterpenes. Farnesyl pyrophosphate synthase (FPS) and lycopene synthase (PSY1) are critical components of carotenoid biosynthesis. FPS facilitates the transformation of isopentene pyrophosphate (IPP) and dimethylallyl pyrophosphate (DMAPP) into farnesyl pyrophosphate (FPP), whereas PSY1 employs FPP as a substrate to catalyze the synthesis of lycopene.

In summary, the FPS family proteins serve as the central hub of isoprene metabolism. Their functions are heavily reliant on the availability of upstream precursors, including HMGS, MK, and IPP2, which influence the flow direction of downstream metabolic pathways such as those leading to sterols, diterpenes, and carotenoids. Additionally, by synergizing with enzymes, such as SQS, DPS, and PSY1, FPS proteins play a crucial role in plant growth, development, and metabolic resistance (Figure 7B).

### 2.6. Analysis of Cis-Acting Elements in the Promoter of EhFPS

**Figure 8:**
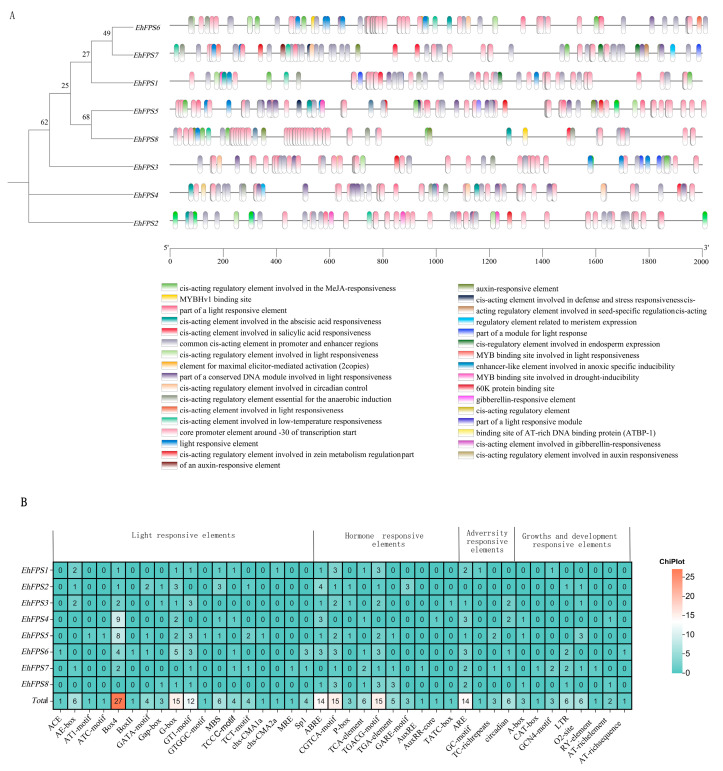
The cis-element in the promoters of *EhFPS* genes. (**A**) Prediction of cis-acting elements in the *FPS* gene promoter region of *E. hirta*. The distribution of 33 cis-acting elements with key functions. (**B**) Statistics of the numbers of cis-elements on the promoters. The various numbers represent the numbers of cis-acting elements.

To understand the expression regulation mode of the *FPS* genes in *E. hirta*, the cis-acting elements of the 2000 bp sequence upstream of the *EhFPS* gene promoter were analyzed (Figure 8). The promoters of the eight *EhFPS* genes contained a large number of cis-acting elements related to the light response, plant hormone induction, and stress response. Statistics for all cis-elements revealed that the *EhFPS* promoter sequence contains 1–27 light-responsive and regulatory elements, which mainly include Box4, G-Box, and GT1-motif elements. Among them, 87.5% of the members had Box4 and G-box elements, and in the hormone response module, they mainly included the jasmonate response elements CGTCA-motif and TGACG-motif and the abscisic acid response element (ABRE). Among them, 30 jasmonic acid (JA) response elements were enriched. Except for the *EhFPS4* promoter, which did not detect the response element of this hormone, the other promoters contained it to varying degrees. Antioxidant response elements (AREs) were significantly enriched in the stress response module, and this element was not detected only in the promoter of *EhFPS2* among the members. In summary, *EhFPS* plays an important role in biological processes such as light induction, hormone induction, and adversity stress response in *E. hirta.*

### 2.7. EhFPS Genes Responds to Exogenous SA and JA Signals

**Figure 9:**
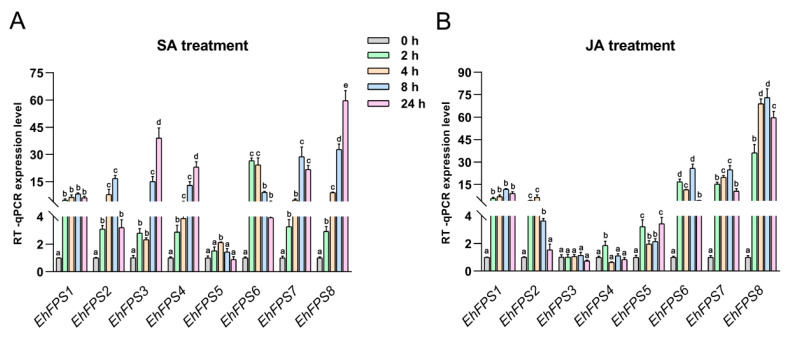
Expression analysis of *EhFPS* genes under SA and JA treatment. (**A**) The gene expression level after SA treatment. (**B**) The expression level of genes after JA treatment. One-way ANOVA and Tukey’s multiple comparison test were used to evaluate the statistical significance. The relative qRT-PCR expression level is shown on the left y-axis. The difference between different letters in the same group was significant at *p* < 0.05. The error bar represents the standard deviation of three biological replicates. Differently colored tiles represent time scale.

Thirty cis-elements that responded to JA signals and six that responded to salicylic acid (SA) signals were detected in the promoters of the eight *EhFPS* genes (Figure 8). Therefore, we tested the expression levels of eight *EhFPS* genes treated with 1.0 mM SA and JA. The results indicated that all *EhFPS* genes, except *EhFPS5*, responded significantly to SA signals with markedly upregulated expression levels (*FC* ≥ 3, *Q* value ≤ 0.01) (Figure 9). Based on the expression trends, *EhFPS* genes were divided into three categories under SA treatment (Figure 9A). Group I included *EhFPS1*, *EhFPS2*, and *EhFPS7*, all of which exhibited a “rise-then-fall” expression pattern. Group II, comprising EhFPS3, *EhFPS4*, and *EhFPS8*, exhibited a gradual increase in expression over time. Group III contained only *EhFPS6* and showed no significant changes, whereas the other family members exhibited an upregulation trend. Under JA treatment, *EhFPS1*, *EhFPS2*, *EhFPS7*, and *EhFPS8* demonstrated a similar “rise-then-fall” expression pattern, with *EhFPS2* peaking at 4 h and the other members peaking at 8 h (Figure 9B). *EhFPS5* did not respond to signals associated with salicylic acid (SA) processing, whereas *EhFPS3* and *EhFPS4* did not respond to signals related to jasmonic acid (JA) processing. In summary, *EhFPS1, EhFPS2, EhFPS7*, and *EhFPS8* responded rapidly and strongly to SA and JA signals, suggesting that they are critically important for terpenoid biosynthesis.

### 2.8. Expression Analysis of EhFPS

**Figure 10:**
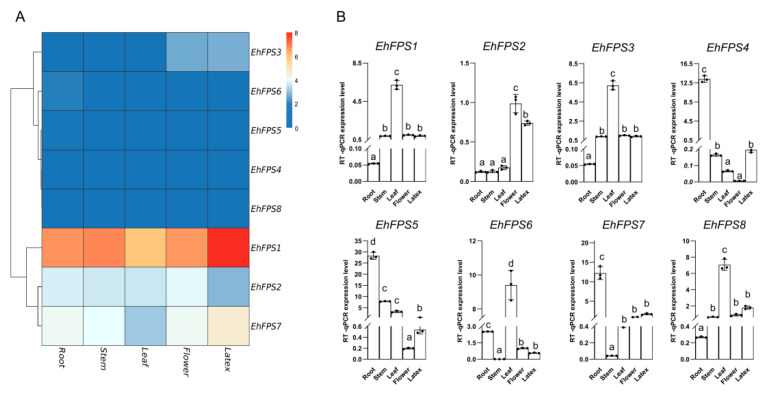
Expression patterns of *EhFPS* in different tissues of *E. hirta*. (**A**) Heat map of transcriptome based on FPKM values. (**B**) Expression profile of eight genes based on average of five tissues. a > 0.05, 0.05 > b > 0.01, 0.01 > c > 0.001, 0.001 > d > 0.0001.

Gene expression is a critical determinant of gene functions. To study the function of FPS in the sesquiterpenes and triterpenes of *E. hirta*, we selected five *E. hirta* tissue samples for transcriptomic analysis. Subsequently, we calculated the average of the expression results and constructed a heatmap to show the expression levels (Figure 10A). RT-qPCR analysis showed *EhFPS* gene-specific expression patterns. *EhFPS1, EhFPS2, EhFPS3*, and *EhFPS5* were highly expressed in the roots, stems, leaves, and flowers, whereas *EhFPS1, EhFPS2, EhFPS3*, and *EhFPS7* were highly expressed in the latex (Figure 10B). The transcriptome was found to be roughly consistent with the quantitative experimental results, and genes with high expression in latex were further screened, namely *EhFPS1, EhFPS2,* and *EhFPS7*. In summary, *EhFPS1, EhFPS2,* and *EhFPS7* from the eight genes were screened for subsequent functional analyses.

### 2.9. Subcellular Localization of EhFPS1, EhFPS2, and EhFPS7

**Figure 11:**
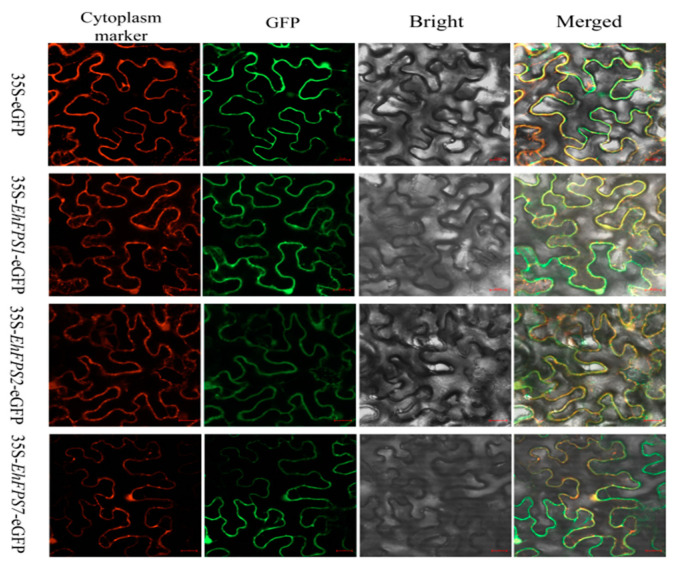
The subcellular localization of EhFPS-GFP fusion protein was transiently expressed in tobacco leaves. Images were captured using a confocal microscope. Control group: 35S-eGFP:35S-pCAMBIA2300-eGFP; cytoplasm marker: 35S-Os1sSrke-RFP, 35S-*EhFPS1*-eGFP, 35-*EhFPS2*-eGFP, and 35S-*EhFPS7*-eGFP were constructed into the 35S-pCAMBIA2300-eGFP vector by homologous recombination of *EhFPS1, EhFPS2*, and *EhFPS7*, respectively. The images include the cytoplasmic marker as a red fluorescence channel (first panels) and GFP as a green fluorescence channel (second panels). The bright-field (third panel) and merged (fourth panel) images are shown on the right. Bar =  20 μm.

The leaves of *N. benthamiana* were infected with the empty pCAMBIA2300-35S-eGFP(C) vector as the control and the experimental group fusion expression vectors pCAMBIA2300-*EhFPS1*-eGFP, pCAMBIA2300-*EhFPS2*-eGFP, and pCAMBIA2300-*EhFPS7*-eGFP. The transformed *N. benthamiana* were placed in a 26 °C incubator under weak light for 2 d, and the green fluorescence and cytoplasmic markers were observed under the 488 nm and 600 nm channels of a laser confocal scanning microscope. The results indicated that the empty pCAMBIA2300-35S-eGFP construct (C) exhibited green fluorescence signals in the nucleus, cytoplasm, cell membrane, and cell wall. In contrast, the GFP fluorescence of the fusion proteins pCAMBIA2300-*EhFPS1*-eGFP, pCAMBIA2300-*EhFPS2*-eGFP, and pCAMBIA2300-*EhFPS7*-eGFP overlapped with the fluorescence of the cytoplasmic RFP protein, which differed from the continuously distributed green fluorescence observed on the cell membrane and cell wall as well as the uniform distribution in the cytoplasm (Figure 11).

## 3. Discussion

Triterpenoids are a class of natural products formed by condensation of isoprene as the basic unit. They are widely found in medicinal plants such as licorice, ginseng, and eleuthero and have significant anti-inflammatory, antibacterial, and antitumor properties. To date, researchers have identified six triterpenoids in *E. hirta*: 3β-taraxacum, taraxerone, tirucallol, cycloart-23-ene-3β,25-diol, 3β-9,19-cyclolanost-23-ene-3,25-diol, and vomifoliol [15]. The *FPS* gene family serves as a crucial rate-limiting enzyme in the terpene synthesis pathway in plants and significantly contributes to the production of hemiterpenes and triterpenoids [16]. Its members have been identified in various medicinal plants and have been shown to play important roles in the synthesis of isoprene and triterpene saponins. The mRNA level of dammarane synthase in *Centella asiatica* was significantly elevated in a similar study that examined the heterologous expression of the *PgFPS* gene cloned from ginseng roots in *C. asiatica* hairy roots. This increased production of saponins and triterpenes in *C. asiatica* results in the rapid accumulation of madecassoside [17]. *FPS* plays a crucial role in the synthesis of bioactive compounds essential for growth and stress responses in plant species such as *Matricaria recutita* [18] and *Blumea balsamifera* [19]. Significant conservation of the *FPS* sequence across plant families, particularly within Asteraceae, was indicated by the discovery of cDNA for *FPS* in various species through sequencing studies. Consistently with our findings, another study reported that the full-length cDNA of the *FPS* gene (*CnFPPS*) in *Chamaemelum nobile* was 1239 bp long and encoded for a 342 amino acid with a molecular weight of approximately 39.38 kDa [20].

An investigation of the sesquiterpene lactone drug artemisinin revealed that overexpression of the endogenous *FPS* gene in *Artemisia annua* can increase endogenous artemisinin content by 2 to 25 times [21]. The simultaneous transfer of two copies of the *FPS* gene reduces artemisinin production in transgenic plants [22]. However, cotransformation of *A. annua* with the *FPS* and *DBR2* genes can significantly increase artemisinin content by 3.36 times [23]. Compared with the wild type, the contents of four ginsenosides, Rh1, Rg1, Re, and Rd, detected in the *FPS* gene overexpression lines of *Panax notoginseng* were significantly increased by 2.66, 1.76, 4.35, and 2.90 times, respectively [24]. However, the identification and expression analysis of *FPS* genes in *E. hirta* have not yet been reported.

In this study, using comparative genomic methods, eight *EhFPS* genes were successfully identified and were found to be located on chromosomes 1, 3, 6, and 8, with chromosome 6 containing five of these genes. Notably, a tandem gene cluster comprising *EhFPS4* and *EhFPS7* was identified. Furthermore, the results of the gene duplication analysis suggest that the evolution of these genes may have resulted in homologous gene pairs through tandem duplication and fragment duplication events, further confirming the evolutionary mechanism of *FPS* genes in plant genomes. In addition, gene duplication analysis results suggest that the evolution of these genes may have been formed by tandem duplication and fragment duplication events. Through comparative analysis, we found that the functional domains of EhFPS protein family members were highly conserved and contained DDXXD, GQXXD, and GKLNR domains that regulate enzyme activities. Previous studies have shown that plant FPPS have five conserved domains like those of other organisms, namely domains I (GKXXR), II (EXXXXXXLXXDDXXDXXXXRRG), III (GQXXD), IV (KT), and VI (GXXFQXXDDXXDXXXXXXXXGKXXXDXXXXK) [25].

Furthermore, it has been found that FPS contains two aspartate-rich regions, namely the DDXXD motifs in domains II and V. This motif plays a crucial role in the catalytic activity of FPS, which is responsible for binding magnesium ions (Mg^2+^), thereby stabilizing pyrophosphate groups and promoting substrate binding and reaction processes. The amino acid residues in this region participate in the formation of C-C bonds between IPP and allylic substrates and determine the chain length of the product. The aspartate and arginine residues in domain II are essential for FPPS function, and their substitution can lead to a decrease in the Vmax of IPP and geranyl pyrophosphate (GPP) while leaving Km largely unchanged. Conversely, mutation of the first aspartate residue in domain V significantly reduced FPS activity and increased the Km value for IPP, confirming the critical role of aspartate in enzymatic activity [26].

In our study, EhFPS8 lacked the DDXXD motif in the conserved domain II, suggesting a weakening of its core catalytic function. However, further experimental validation is required. Through physical and chemical property analyses, EhFPS proteins were found to be hydrophilic and have a high proportion of α-helices. This is similar to the FPS proteins reported in rubber trees and other plants, the α-helical structures of which are often closely related to their catalytic activity and substrate binding ability [27,28]. Previous studies have shown that prerequisite C5 units (DMAPP and IPP) are synthesized through both the plastidial methyl-D-erythritol phosphate (MEP) and cytosolic MVA pathway.

However, the subsequent biosynthetic pathways for different terpenoid classes vary [29]. Specifically, sesquiterpenes and triterpenes are synthesized via the cytosolic MVA pathway, whereas monoterpenes, diterpenes, and tetraterpenes are synthesized through the plastidial MEP pathway [30]. For example, cytoplasmic localized *AtFPS1S* can participate in the synthesis of sesquiterpenes, such as (+)-δ-cadinene, and in inducible defense against aphid infestation in *Arabidopsis* [31]. In ginseng, *PcFPS* localized in the cytosol is capable of regulating triterpenoids such as pachymic acid and eburicoic acid [32]. *AtFPS2* encodes an FPS enzyme that localizes to the chloroplast. This enzyme is involved in the synthesis of monoterpenes and diterpenes, which are essential components of chlorophylls, carotenoids, and gibberellins.

In this study, previous predictions using subcellular localization showed that *EhFPS1, EhFPS2, EhFPS5, EhFPS6*, and *EhFPS7* were localized in the cytoplasm, whereas *EhFPS3, EhFPS4*, and *EhFPS8* were localized in the chloroplasts. In addition, the subcellular localizations of *EhFPS1, EhFPS2*, and *EhFPS7* proteins in *N. benthamiana* were confirmed (Figure 11). Subcellular localization prediction analysis indicated that, except for *EhFPS3, EhFPS4*, and *EhFPS8*, the remaining EhFPS proteins were primarily localized in the cytoplasm. This finding is consistent with their role in terpenoid biosynthesis, since most terpenoid synthases are active in the cytoplasm. In contrast, those localized in the chloroplast, such as *EhFPS3, EhFPS4,* and *EhFPS8*, may play roles in the metabolic pathways associated with photosynthesis in plants. In this study, GO enrichment analysis revealed that *EhFPS* genes were significantly enriched in biological processes related to isoprenoid metabolism and lipid biosynthesis, indicating that these genes play crucial roles in the secondary metabolism of *E. hirta*. This finding aligns well with our hypothesis regarding the function of the FPS protein family, suggesting their important role in the synthesis of isoprenoids and lipid compounds. Previous studies have demonstrated that *FPS* genes play a pivotal role in isoprenoid metabolism in plants, particularly in the biosynthesis of terpenoids and carotenoids [18,19,20,32]. Moreover, comparative analysis showed that the functional domains of *EhFPS* genes are highly conserved, including the enzyme activity-regulating motifs DDXXD and GKLNR, further supporting their hypothesized functions as terpene synthases. The type and number of cis-acting elements in the promoter region play important regulatory roles in gene expression [33].

The promoter of the *FPS* gene of *E. hirta* contains a large number of cis-acting elements related to light response, plant hormone induction, and stress response, indicating that members of this family are involved in a variety of biological processes. Studies have reported that spectral quality mainly changes the distribution of terpenoids, whereas the intensity and period affect their abundance [34]. For example, illumination duration and temperature have an important impact on the growth of *Ganoderma lucidum* (a Chinese medicine) and synthesis of triterpenes. The combination of 36 °C and 16 h of illumination significantly increased the yield of *Ganoderma lucidum* triterpenes, which was 98.32% higher than that of the control. Light treatment can also induce an increase in the mycelial biomass of *Ganoderma lucidum* and promote the synthesis of triterpenoids by upregulating the expression of key enzyme genes such as HMGR, SQS, and LAS [35]. Another study on *Eleutherococcus senticosus* revealed that saponin content significantly increased under varying light conditions. Four *EsbZIP* genes associated with saponin biosynthesis were identified through correlation analysis: *EsbZIP1*, *EsbZIP2*, *EsbZIP4*, and *EsbZIP5*. The promoter region of *EsbZIP* contains numerous photoresponsive elements, and its activity varies with light intensity [36].

We observed that the promoters of the eight *EhFPS* genes contained different numbers of light-responsive cis-elements (Box4, G-box, and GT1-motif). Therefore, it can be speculated that the transcription level of *EhFPS* may be regulated by transcription factors involved in terpene synthesis, such as AP2/ERF, BHLH, MYB, NAC, WRKY, and bZIP, which ultimately affect the synthesis and accumulation of triterpenoids [37]. Plant hormones have been reported to affect terpenoid synthesis through various mechanisms. For example, plant hormones such as JA, MeJA, and SA can regulate the differential expression of key enzyme activities or genes (HMRG, DXR, and FPS) in the terpenoid biosynthesis pathway by acting as signaling molecules or inducers, thereby affecting the accumulation of the final product [38]. Moreover, plant hormones, such as GA, BR, and ABA, are also involved in regulating the synthesis of terpenoids [39,40]. Seven genes linked to ginsenoside Rg3 synthesis (FPS, UGT, CYP339, etc.) were screened using trait correlation analysis. Exogenous MeJA treatment markedly increased the expression levels. Six hours after induction, the expression level of the *FPS* gene peaked and was 7.1 times higher than that in the control group [41]. It has been discovered that treating birch trees with exogenous MeJA at a concentration of 0.5 mmol/L and SA at a concentration of 50 mmol/L can greatly raise the *BpFPS* gene’s expression level and encourage the buildup of total triterpenoids in the leaves and outer bark of the stem [42].

In addition, promoter analysis and RT-qPCR quantitative experiments revealed that the promoters of birch *BpMYB21* and *BpMYB61* contain plant hormone response elements, including ABRE, CGTCA-motif, TCA-element, and GARE motifs. These elements can be induced by ABA, JA, and SA and subsequently control the production of triterpenoids by regulating the expression of important enzyme genes (SS and SE) [43]. A MeJA response element was found in the promoter sequence of the *CbWRKY24* gene of *Ursula bile*, which responds to MeJA induction. In overexpressed plants, it was found that the expression levels of genes such as *HMGR*, *FPS*, *SE*, and *β-AS* were significantly upregulated, thereby improving the total saponin content of bear bile grass [44]. Treatment of *Panax notoginseng* with three plant elicitors (JA, ABA, and SA) can upregulate the expression levels of the key genes *PgFPS* and *PgIPPI* for terpenoid synthesis, promote the synthesis of triterpenoid compounds, and promote the accumulation of triterpenoid saponins [45]. Gene expression analysis of *M. recutita* revealed that *MrFPS* was significantly upregulated in response to methyl jasmonate. The expression patterns varied considerably among different plant tissues, with the highest expression levels observed in flowers and stems. This tissue-specific distribution implies that different plant sections have distinct roles for the FPS enzyme in the production of secondary metabolites [18]. *CnFPPS* expression was consistently observed across various tissues in *C. nobile*, with the highest concentrations found in the root system. This distribution pattern indicated that *CnFPPS* may play a key role in regulating terpenoid production [20]. Functional analyses of *AvFPS* revealed that *Aconitum vilmorinianum* was significantly upregulated in response to methyl jasmonate. Moreover, genetically modified expression of *AvFPS* increased the plant’s resistance to environmental stressors, which increases its ability to be cultivated under less ideal circumstances [46].

In summary, plant hormones affect the biosynthesis and accumulation of triterpenoid compounds by affecting key enzyme activities, differential expression of key enzyme genes, regulation of transcription factors and promoter activities, and synergistic interactions between plant hormones [32]. In this study, a large number of cis-elements (ABRE, TCA-element, CGTCA-motif, and TGACG-motif) that responded to the plant hormones ABA, SA, and MeJA were also found in the promoters of *E. hirta FPS* genes. Through exogenous JA and SA treatments, we further investigated the expression patterns of *EhFPS* genes under SA and JA signaling. It was found that, except for *EhFPS5*, all other genes responded to SA treatment, exhibiting significant upregulation of expression. The expression trends of *EhFPS* genes could be divided into three subgroups, with *EhFPS1, EhFPS2*, and *EhFPS7* showing similar expression patterns, whereas *EhFPS3, EhFPS4*, and *EhFPS8* displayed a consistent trend. This is consistent with the known relationship between terpenoid biosynthesis and hormone signaling in plants. Under exogenous JA treatment, genes such as *EhFPS1, EhFPS2, EhFPS6, EhFPS7*, and *EhFPS8* rapidly responded to JA signals, showing a significant increase in expression levels.

We also observed that some genes, such as *EhFPS1, EhFPS2*, and *EhFPS7*, responded quickly to both SA and JA signals, demonstrating expression patterns similar to those of terpenoid synthases under various stress conditions, as reported in the literature [39]. This result is also consistent with our findings from cis-acting element analysis of the promoters. These experiments further support the hypothesis that these genes play a key role in terpenoid biosynthesis. Therefore, it is speculated that terpenoid compounds are mainly synthesized in the latex and leaves. Terpenoids are widely present in nature and constitute the main components of essences, resins, and pigments in some plants [47]. Tissue-specific expression analysis revealed that *EhFPS1, EhFPS2*, and *EhFPS7* exhibited significantly higher expression levels in the latex of *E. hirta* than the other genes, suggesting that these genes may be involved in the regulatory pathway of latex synthesis. This finding was consistent with our hypothesis regarding terpenoid biosynthesis. Natural rubber is a natural polymer composed primarily of cis-1,4-polyisoprene, which belongs to the terpenoid class and is a major component of resins. Specific expression of these genes in latex is crucial for terpenoid biosynthesis. Similar tissue-specific expression patterns have been reported in rubber trees and other plants, further supporting our understanding of the functional roles of *EhFPS* genes [48]. Importantly, the FPS gene family is essential for metabolic activities that produce different terpenoids, which serve as defenses against infections and organisms that consume plants. For example, the *FPS* found in *M. recutita* contributes to the synthesis of α-bisabolol, a pharmacologically active substance essential for its medicinal effects [18]. Investigation of the *FPS* gene family in several species demonstrated its essential function in plant metabolism, specifically in the biosynthesis of terpenoids, which are essential for development, growth, and stress reactions [18,19,20]. The need for further research to fully understand the biological importance of *FPS* genes is underscored by their high degree of conservation, diverse expression patterns, and functional implications for secondary metabolism.

## 4. Materials and Methods

### 4.1. Experimental Materials

Wild-type *E. hirta* used in this study was provided by the Chinese Academy of Tropical Agricultural Sciences, Haikou, Hainan Province, China (110°33′ E,19°98′ N). We selected 20 samples of *E. hirta* at the same growth stage, cleaned the plant surfaces with 0.1 M PBS buffer, and subsequently separated the root, stem, leaves, flowers, latex, and other tissue samples while maintaining low temperatures. Equal amounts of these mixed samples were stored on dry ice and sent to Shanghai Ouyi Biotechnology Co, Ltd. (Shanghai, China) for transcriptome sequencing. The wild-type *E. hirta* plants utilized in this study exhibited uniform growth 45 days after sowing. Indoor culture conditions were maintained at 25 °C with a light intensity of 400 μmol m^−2^ s^−1^ for 14 h, followed by 8 h of darkness. We weighed 0.021 g of jasmonic acid (JA) and 0.0138 g of salicylic acid (SA) as dry powders, which were then dissolved in anhydrous ethanol. The solution was titrated with double-distilled water (ddH_2_O) to achieve a final volume of 1 L, with the pH adjusted to 6.8. This procedure resulted in a working solution of JA and SA at concentration of 0.1 mM. To prevent degradation, it is crucial to avoid exposure to light and to store the solution at 4 °C. In a low-light environment, the solution was sprayed onto both surfaces of the plant leaves at a distance of 20 cm. The treatments were applied at 0, 2, 4, 8, and 24 h, with three biological replicates established at each time point. *N. benthamiana* used in the subcellular localization experiment was provided by the Molecular Breeding Laboratory of the Rubber Research Institute, Chinese Academy of Tropical Agricultural Sciences, Haikou, Hainan Province, China.

### 4.2. Identification and Physicochemical Analysis of EhFPS Genes

The domains contained in the rubber tree *FPS* gene were analyzed using CDS-Search (https://www.ncbi.nlm.nih.gov/Structure/cdd/cdd.shtml, accessed on 7 May 2024) and InterPro (https://www.ebi.ac.uk/interpro/ accessed on 7 May 2024). FPS family members were identified using BLASTP of the genome and transcriptome data of *E. hirta* (unpublished) and named according to their physical distance. The chromosomal location of *EhFPS* genes and identification of tandemly repeated genes were determined using a previously described method [49]. The *E. hirta* GFF annotation file was input into the Gene Location Visualization from the GTF/GFF program of TBtools-II v. 2.136 software, which was used to obtain a rough distribution map of *EhFPS* genes on chromosomes. Referring to the criteria for determining gene clusters, two adjacent genes were no more than 250 kb apart on the chromosome, the tandemly arranged *FPS* genes on each chromosome were identified, and their Ka/Ks were calculated. The online tool ExPASy ProtParam (https://web.expasy.org/protparam/ accessed on 17 July 2024) was used to predict and analyze the physicochemical indices of *EhFPS* genes, including amino acid molecular weight, protein length, theoretical isoelectric point, α-helix, and β-turn. The online tool SWISS-MODEL (https://swissmodel.expasy.org/interactive accessed on 18 June 2024) was used to predict the secondary structure of the *EhFPS* genes. The WOLF PSORT website (https://wolfpsort.hgc.jp/ accessed on 19 July 2024) was used to predict the subcellular localization of the *EhFPS* genes.

### 4.3. Phylogenetic Analysis

The amino acid sequences of *EhFPS* genes were added to the NCBI database (https://www.ncbi.nlm.nih.gov/ accessed on 21 July 2024), and the highly homologous FPS protein sequences in the rubber tree, cassava, *Eucommia*, dandelion, and *A. thaliana* were retrieved and downloaded through BLASTP comparison (“1 × 10^−5^”, “1 × 10^−10^”). The biological software Clustalw v. 1.83 was used to perform a complete multiple sequence alignment of the screened amino acid sequences, and a phylogenetic tree was constructed using the neighbor-joining method (bootstrap value was set to 1000) in MEGA v. 7.0. Optimization was performed using the online software Evolview v. 4 (https://www.evolgenius.info/evolview accessed on 21 July 2024).

### 4.4. Gene Structural Analysis of EhFPS

The MEME online tool (https://meme-suite.org/meme/tools/meme accessed on 22 June 2024), NCBI (https://www.ncbi.nlm.nih.gov/Structure/cdd/wrpsb.cgi accessed on 22 June 2024), and the InterPro database (https://www.ebi.ac.uk/interpro/ accessed on 22 June 2024) were used to analyze the conserved motifs of proteins, with the base number set to 10 and other parameters as default. The exon–intron structure of *EhFPS* was analyzed using TBtools-II v. 2.119 [50], and the phylogenetic tree, conserved domains, and gene structures were visualized.

### 4.5. Collinearity Analysis and Visualization of FPS Genes Between H. brasiliensis and E. hirta

Referring to the experimental method of Xue et al. [51], a script was used to extract the information files required by TBtools such as chromosome length, chromosome gene information, and link information between chromosomes based on collinearity and GFF annotation files. The files were then input into the Advanced Circus plug-in TBtools to complete the collinearity analysis between *E. hirta* and *H. brasiliensis*, and to draw a collinearity map between chromosomes.

### 4.6. Cis-Acting Elements Analysis in the Promoter of EhFPS

The plug-in Fasta extract in TBtools was used to extract the 2000 bp sequence upstream of the gene transcription start site as the promoter analysis region. The cis-acting elements were predicted using the promoter analysis online website PlantCare (https://bioinformatics.psb.ugent.be/webtools/plantcare/html/ accessed on 21 June 2024), and the prediction results were visualized using the plug-in Basic Bio-sequence View.

### 4.7. Expression Pattern Analysis

Transcriptome data from our laboratory were used to analyze the expression levels of the *FPS* gene family members in different tissues of *E. hirta*. The transcriptome data obtained by sequencing were uploaded to the local server (SSH-2.0-OpenSSH_7.4:10.38.0.1) the RSEM software v. 2.1 package was used for expression analysis to obtain the FPKM value of each gene, and the HeatMap plugin in TBtools was used to draw a heat map.

### 4.8. Gene Ontology Enrichment Analysis

Gene ontology (GO) enrichment analysis was performed to understand the biological roles of the identified genes. Functional annotations were derived from molecular breeding laboratory databases, providing a foundation for categorizing genes based on their biological processes, molecular functions, and cellular components. The analysis was conducted using the Cluster Profiler R package v. 4.14.0, which facilitated systematic identification of overrepresented GO terms among the target gene sets. Statistical significance was determined using thresholds of *Q* < 0.05 and *p* < 0.05. Visualization of enriched GO terms was performed using the same package.

### 4.9. Interaction Network of E. Hirta Homologues in A. Thaliana

Homologous genes of *E. hirta* were identified in *A. thaliana* using OrthoVeen v. 3.0 (https://orthovenn3.bioinfotoolkits.net/ accessed on 21 November 2024). Further analysis of the *EhFPS* homologous gene set using STRING (https://cn.string-db.org/ accessed on 21 November 2024) with a minimum interaction score of 0.6 was required to limit the results to no more than 30 interactors. Genes with homologues in *E. hirta* were compared, filtered from the interaction results, and visualized using bioinformatics analysis performed using OECloud tools.

### 4.10. RNA Extraction and RT-qPCR Analysis

Reverse transcription quantitative PCR validation and expression analysis of *FPS* genes were performed using transcriptome data from *E. hirta*. Total RNA from five tissues (root, stem, leaf, flower, and latex) of wild-type *E. hirta* was extracted using a SteadyPure Plant RNA Extraction Kit (AG21019; Aikerui, Changsha, China). The concentration and integrity of the RNA samples were determined using a NanoDrop 2000 spectrophotometer and gel electrophoresis. cDNA was synthesized by reverse transcription using the PrimeScript™ RT Reagent Kit and the gDNA Eraser Kit (Takara, Dalian, China). RT-qPCR primers for *EhFPS* genes were designed using the online Integrated DNA Technologies website (https://www.idtdna.com/scitools/Applications/RealTimePCR/default.aspx accessed on 21 November 2024) (Table S3). RT-qPCR was performed using *ACT1.2373* as the internal reference gene and the reverse-transcribed cDNA obtained above as the template, according to the instructions of the 2 × Q3 SYBR qPCR Master Mix (Universal) kit (2205, TOLOBIO, Shanghai, China). The relative expression level of *EhFPS* was calculated using the 2^−ΔΔCt^ method, and the results were plotted using the data statistics software Prism v. 10.

### 4.11. Cloning of CDS Sequences of EhFPS1, EhFPS2, and EhFPS7 and Construction of Fusion Expression Vector

Total RNA was extracted from wild-type *E. hirta* leaves, and its concentration and integrity were determined using a NanoDrop 2000 spectrophotometer and gel electrophoresis. The extracted total RNA sample was analyzed by electrophoresis, which clearly showed two bands, 28S and 18S, indicating that the extracted total RNA was of good quality and met the requirements for reverse transcription. Agarose gel electrophoresis (0.8%) was used to detect the linearized vector and the CDS sequence amplification products of *EhFPS1*, *EhFPS3*, and *EhFPS7* (Figure S1A). The empty pCAMBIA2300-35S-eGFP(C) plasmid was a single band with a size of approximately 10,233 bp, while the electrophoretic migration rate of the pCAMBIA2300-35S-eGFP(C) double enzyme digestion product was significantly lower than that of the circular plasmid. Specific primers designed according to the CDS region were used for amplification, and the product sizes of *FPS1*, *FPS3*, and *FPS7* were target bands of 1029, 1053, and 1011 bp, respectively (Figure S1B). Single clones of the recombinant plasmids were selected from *E. coli* for PCR identification. The results showed that the PCR products of the two selected single clones contained single bands of 1029, 1053, and 1011 bp, confirming that they were both positive (Figure S1C), and were subsequently sent to Shanghai Sangon Biotechnology Co., Ltd. (Shanghai, China) for Sanger sequencing.

### 4.12. Transient Expression of EhFPS1, EhFPS2, and EhFPS

According to the CDS sequences of *EhFPS1*, *EhFPS3*, and *EhFPS7*, CE Design v. 1.04 software was used to design specific amplification primers for the homology arms of the vector using BamHI and SalI restriction sites (Table S4). The CDS sequences of *EhFPS1, EhFPS2,* and *EhFPS7* were amplified using reverse-transcribed cDNA as a template. The system was as follows: Primer-F 0.5 μL, Primer-R 0.5 μL, cDNA 1 μL (200 ng/μL); TakaRa 2 × PrimeSTAR^®^ Mix 25 μL, ddH_2_O 23 μL. Amplification program: 98 °C/3 min, 98 °C/10 s, 65 °C/10 s, 72 °C/1 min (35 cycles), 72 °C/1 min, stored at 4 °C. The plasmid was then recombined and transformed into DH5α competent cells. Following successful sequencing, the recombinant plasmid was transformed into GV3101-p19 competent cells in accordance with Zhao’s method [52]. The transformed cells were then used to infect *Nicotiana benthamiana*, which were maintained in a dark culture room at 26 °C for 48 h. Observations were made and recorded using a laser confocal microscope (ZEISS LSM800, Göttingen, Germany) under a 488 nm channel.

## 5. Conclusions

This study employed the genome and transcriptome data of *E. hirta* and systematically identified eight *FPS* genes distributed on chromosomes 1, 3, 6, and 8. Through the identification of conserved domains and phylogenetic analysis, it was found that seven family members contained DDIMD motifs, all of which contained GQMXD and DDYXD motifs. The gene structure was highly conserved and the relationship was close. In the promoter region of *EhFPS* genes, we identified large numbers of light-responsive elements (Box4, G-box, and GT1-motif) and hormone-responsive elements (ABRE, CGTCA-motif, TGACG-motif, and TCA-element) and verified whether their expression levels were induced by relevant hormones. In addition, expression pattern analysis revealed that *EhFPS1, EhFPS2*, and *EhFPS7* were expressed specifically in various tissues and their encoded proteins were localized in the cytoplasm. These results provide a theoretical basis for in-depth research into the regulatory mechanisms and biological functions of *EhFPS* genes in the synthesis of terpenoids in *E. hirta*.

## Figures and Tables

**Table 1:** The basic information of *EhFPS* genes.

Gene Name	No. of Amino Acids	Molecular Weight (Kda)	Isoelectric Point (PL)	Total Average Hydrophobicity	Secondary Structure (%)				Subcellular Localization
					Alpha Helix (%)	Beta Turn (%)	Extendstrand (%)	Randomcoli (%)	
*EhFPS1*	342	39.52	5.5	−0.264	63.16	2.92	7.02	26.90	Cytoplasm
*EhFPS2*	350	40.50	5.11	−0.255	65.43	3.14	5.71	25.71	Cytoplasm
*EhFPS3*	389	45.24	5.91	−0.305	59.90	3.34	6.17	30.59	Chloroplast
*EhFPS4*	335	38.64	5.96	−0.348	56.72	2.99	8.96	31.34	Chloroplast
*EhFPS5*	323	37.44	5.14	−0.293	65.94	2.48	7.74	23.84	Cytoplasm
*EhFPS6*	342	39.73	5.49	−0.353	65.50	2.92	6.43	25.15	Cytoplasm
*EhFPS7*	336	38.97	6.52	−0.317	63.99	2.98	7.14	25.89	Cytoplasm
*EhFPS8*	346	39.95	6.52	−0.323	61.27	3.76	5.49	29.48	Chloroplast

## Data Availability

All analyzed data can be found in the article or in the Supplementary Material.

## Supplementary Materials

The following supporting information can be downloaded at: https://www.mdpi.com/article/10.3390/ijms26020798/s1, Figure S1: Gene amplification and vector construction. (A) RNA detection; (B) 35::2300::eGFP vector linearization and amplification of EhFPS1, EhFPS2 and EhFPS7 genes; (C) Colony PCR verification, 1–5: recombinant EhFPS1, 6–10: recombinant EhFPS2, 11–15: recombinant EhFPS3. Bands are shown at specific length above 10,000 bp and 1000 bp; Table S1: Identified genes under gene ontology enrichment analysis; Table S2: FPS proteins homologues and their interaction network with other proteins; Table S3: List of primers used for qRT-PCR; Table S4: List of fusion expression vector primers.
